# Epidemiology of suicide and suicide attempts in Jahrom district, Southern Iran in light of COVID pandemic: A prospective observational study

**DOI:** 10.1002/hsr2.933

**Published:** 2022-11-14

**Authors:** Mina Sefid Fard Jahromi, Mohammad Hadi Eghbal, Vahid Rahmanian

**Affiliations:** ^1^ Psychiatry, Research Center for Non‐Communicable Diseases Jahrom University of Medical Sciences Jahrom Iran; ^2^ Faculty of Medicine, Research Center for Social Determinants of Health Jahrom University of Medical Sciences Jahrom Iran; ^3^ Epidemiology, Research Center for Social Determinants of Health Jahrom University of Medical Sciences Jahrom Iran

**Keywords:** COVID‐19, depression, mental disorders, outbreak, suicide

## Abstract

**Background and Aims:**

Suicide is a global public health issue. The covid‐19 epidemic has led to disturbance in daily life and economic activity. It is assumed that increased stress and anxiety cause suicide. This study aimed to describe the causes and methods of committing suicide during the COVID pandemic.

**Methods:**

This descriptive study was conducted on all outpatients, inpatients, and those who died during the period from March 21, 2021 to March 20, 2022 due to suicide attempts, referred to hospitals and clinics affiliated with Jahrom University of Medical Sciences or were identified by the social emergency and welfare organization.

**Results:**

A total of 330 suicide attempters with an average age of 26.74 ± 0.64 years were studied. They were 220 women (66.67%), 159 single (48.18%), and 309 (73.64%) people who had diplomas and high school degrees. In general, the three main reasons for committing suicide were emotional issues and problems with 169 people (51.21%), family violence with 127 people (38.48%), and economic issues and problems with 90 people (27.27%). In terms of suicide manners, 283 people (85.76%) used medicine (or pharmaceuticals), 16 people (4.85%) used agricultural pesticides, and 11 people (3.33%) used rodenticides. Furthermore, 164 people (49.70%) suffered from depression, 94 people (28.49%) were children of divorce, 60 people (18.18%) were drug users, 151 people (45.76%) had a history of alcohol consumption, and 116 people (35.15%) had a history of committing suicide. A total of 6 cases of complete suicide (leading to death) have occurred.

**Conclusion:**

The most important factors for suicide throughout the covid‐19 epidemic were emotional issues, marital incompatibility, and economic issues. Medicine poisoning was the most important method of committing suicide. In times of crisis, a multisectoral public health approach is needed to prevent increased anxiety, stress, and subsequent suicide attempts.

## 1 INTRODUCTION

Suicide is a deadly act that shows a person's desire to die.^1^ Given by the World Health Organization (WHO), more than 700,000 persons die via suicide each year, and on average, one person commits suicide every 40 s, and it is the fourth cause of death among people aged 15−29 in the world.^2^, ^3^ The WHO also reports that in the last 45 years, the suicide rate has increased by 60% worldwide.^2^

In general, suicide has been reported in all countries, but 77% of these occur in low‐ and middle‐income nations. The global age‐standardized rate for 2019 was 9 per 100,000 people. This rate is reported to be 12.6 per 100,000 for men and 5.4 per 100,000 for women.^4^

This rate is reported to be 8.1 in South East Asian women as a higher rate compared to the average global in females and 18.1 in men in Africa, 17.1 in Europe, and 14.2 in America per 100,000, a higher rate than the average global in males.^2^, ^4^

In 2019, in the Eastern Mediterranean Region countries of the WHO, Somalia and Djibouti, with age‐standardized rates of 14.7 and 11.9 per 100,000 people, have the highest incidence rates, and Jordan and the Syrian Arab Republic with an incidence rate of 2 and 2.1 per 100,000 people have the lowest rates. Iran ranks tenth among the 21 countries in the region regarding the suicide rate, with an incidence rate of 5.1 per 100,000 people^4^ (Supporting Information: Table 1).

The rate of suicide in Iran in 1979 in the general population was 4.4, in males, 5.7 and females 1.3 per 100,000,^5^ and in 2015 overall was 8, in males 11.1 and females 4.7 per 100,000.^6^

Although the association between suicide and psychiatric disorder has been well established,^7^, ^8^, ^9^ many suicides occur impulsively in moments of crisis.^10^, ^11^ Additional risk factors contain experiencing loss, solitude, prejudice, relationship breakdown, violence, economic difficulties, abuse, chronic diseases, and other conflicts.^2^

According to the results of a study in high and upper‐middle‐income countries, the number of suicides in the early months of the Covid‐19 pandemic was unchanged or decreased compared to the period before the pandemic.^12^ In contrast, some studies shown through the covid‐19 epidemic have described higher rates of suicide in various nations, such as India,^3^, ^13^ Poland,^14^ Bangladesh,^15^ and Norway.^16^ Nevertheless, social, health, political, cultural, and economic scenarios differ from country to country.^11^ The covid‐19 epidemic, the most critical public health problem in the new generation, is due to changes in normal living conditions such as quarantines and long‐term lockdowns, stay‐at‐home policies, and business closures. Its impact on social and economic activities has led people to engage in harmful behaviors, especially among individuals of low socioeconomic class.^17^, ^18^, ^19^, ^20^

The causes of suicide in various countries, cultures, climates, ethnicities, and additional time conditions, especially in crises, due to differences in management control policies, can be different.^21^, ^22^ According to the WHO, only 38 countries have a national strategy for suicide prevention. The death rate for suicide reflects the 3.4 goals of the Sustainable Development Goals via 2030 to decrease premature deaths from noncommunicable diseases by one‐third.^23^

Thus, this study aimed to describe the causes and methods of committing suicide among people referred to hospitals, clinics, and centers affiliated with Jahrom University of Medical Sciences during the COVID‐19 pandemic.

## 2 METHOD

### 2.1 Study design

This is a descriptive (prospective observational) study on 330 outpatients, inpatients, and deceased patients who, from March 21, 2021 to March 20, 2022 were referred to hospitals and clinics affiliated with Jahrom University of Medical Sciences due to suicide attempts or were identified by the social emergency and welfare organization of this city. The inclusion criteria were consent to study, practically attempted suicide, whether this action was successful or not, whether the intention of this action was certain death or scaring others with the goal of gain, and whether the person himself or his companions were able to collaborate with the investigator for interview. Patients poisoned with alcohol or substance were not among those who tried to commit suicide unless they intended to commit suicide.

### 2.2 Data collection

The data collection instrument was a checklist designed according to the objectives and study variables (data collection form) in such a way that during the period of the study, the researcher (physician) was prospectively notified daily in person or by phone about the arrival of new patients to these centers.

Data collection was done by a trained physician based on the study's objectives. The patient was interviewed in Persian, and the relevant checklist was completed.

To determine the instrument's validity, experts' opinions were used, and the checklist questions were examined and approved in terms of relevance to the topic, clarity and simplicity of the literature, and necessity. The reliability of the checklist was confirmed by calculating Cronbach's *α* (0.705).

In cases where the patient died due to suicide, the family members of the death person were interviewed after obtaining oral consent, and the checklist was completed. In addition, to confirm and check the reliability of the data, the physician responsible for data collection reviewed the information from the patient's medical records. If the information reported by the patient did not match the information recorded in the patient's medical records, the issue was raised with the patient‐physician and the nurse in charge of the department. After detailed investigations, the patient or his family were again interviewed.

The studied variables include age, sex, location, marital status, job, education, monthly revenue, history of suicide attempt in the individual, background of suicide attempt in the household, cause of suicide, methods of suicide attempt, drug use, alcohol use, history of mental illness, previous declaration of suicide intention, the time interval between decision and action, and child of divorce. It should be mentioned that in this study, emotional divorce means the existence of many arguments and cold and not intimate relationships between couples. Addiction was defined as the frequent and high consumption of a substance that causes distress and a strong desire to consume that substance again. Therefore, the meaning of alcohol or drug addiction in this study is the definition mentioned above, and consumption of smaller amounts was called recreational consumption.

To measure the history of neurological and mental diseases such: as depression, anxiety, bipolar, schizophrenia, personality disorders, and other physical illnesses, the data recorded in the patient's medical record, which was based on the clinical diagnosis of a Psychiatry specialist, was used.

### 2.3 Statistical analysis

The IBM SPSS software version 22 was used to analyze the data. Descriptive (mean ± SD or frequency and percentage) and analytical (*χ* ^2^ and Fisher exact test) statistics were used. *p* < 0.05 was considered a significant level.

## 3 RESULTS

During the study period, 730 individuals were visited, and 474 met the inclusion criteria. A total of 330 suicide attempters (69.62% participation rate), with an average age of 26.74 ± 0.64 years, were studied. They were 220 women (66.67%), 159 were single (48.18%), 126 were married (38.18%), and 230 (69.70%) were city residents. In terms of education, 309 people (73.64%) were in secondary and high‐school or less, and 113 people (34.24%) had a revenue level of fewer than 3 million tomans each month (Table 1).

**Table 1:** Distribution of demographic characteristics in suicide attempters referring to Jahrom University of Medical Sciences Centers in 2021

Variable	Category	Frequency	Percentage	*p* Value
Age (years)	Under 15	23	6.97	<0.0001
	15−20	89	26.97	
	20−25	60	18.18	
	25−30	53	16.06	
	30−35	34	10.30	
	35−40	26	7.88	
	Over 40 years old	45	13.64	
Gender	Male	110	33.33	<0.0001
	Female	220	66.67	
Marital status	Single	159	41.18	<0.0001
	Married	126	38.185	
	Divorce or death of spouse	45	13.64	
Residence	City	230	69.70	<0.0001
	Village	100	30.30	
Action result	Dead	6	1.82	<0.0001
	Alive	324	98.18	
Education	Primary	87	26.36	<0.0001
	Secondary and high‐school	222	67.27	
	Graduate and above	21	6.37	
Job	unemployed or student	204	61.82	<0.0001
	Government employee	11	3.33	
	Civil servant	3	0.91	
	freelance job	40	12.12	
	Does not have a permanent job	72	21.82	
Monthly income (million tomans)	Under 3	113	34.24	<0.0001
	3−5	87	26.36	
	5−8	76	23.03	
	More than 8	54	16.37	

**Figure 1:**
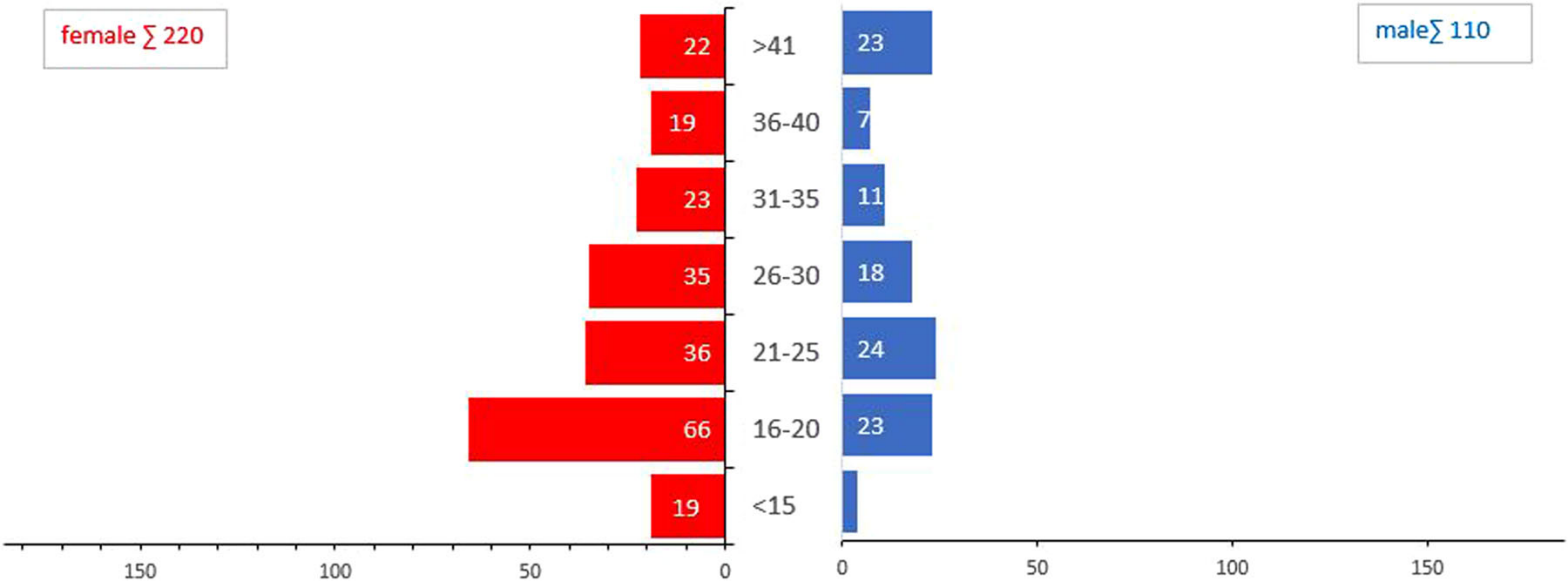
Age‐sex pyramid distribution of suicide attempters referred to centers affiliated with Jahrom University of Medical Sciences in 2021

Among males, the age category of 21−26 years with 24 people (21.8%), and among females, the age category of 16−21 with 66 people (30%) has the highest frequency (Figure 1).

Regarding risk factors, 116 people (35.15%) had a background of suicide attempts, and depression was the most common disease among the studied people, with a number of 164 people (49.70%). Also, 116 people (35.15%) of those who took action had somehow informed others about their decision before their action, and 192 people (58.18%) of them had implemented their decision in less than 24 h. On the other hand, 94 people (28.49%) of those who committed suicide were children of divorce, and 100 people (30.30%) of them reported the existence of emotional divorce between their parents (Table 2).

**Table 2:** High‐risk behaviors in suicide attempters referring to hospitals, clinics, and centers affiliated with Jahrom University of Medical Sciences in 2021

Variable	Category	Frequency	Percentage	*p* Value
History of attempted suicide in a person	First time	214	64.85	<0.0001
	Second time	66	20	
	More than twice	50	15.15	
Family history of suicide attempts	No	183	55.45	<0.0001
	In second‐degree relatives	101	30.61	
	In first‐degree relatives	46	13.94	
History of neurological and mental diseases	I do not have	108	32.73	<0.0001
	Depression	164	49.70	
	Anxiety	111	33.64	
	Bipolar	5	1.52	
	Schizophrenia	4	1.21	
	Personality disorders	49	14.85	
	Other physical diseases	22	6.67	
Under medical treatment	No	235	71.21	<0.0001
	Benzodiazepines	40	12.12	
	Anti‐depressant	32	9.70	
	Antipsychotics	18	5.45	
	Anticonvulsants	22	6.67	
	Lithium	3	0.91	
	Antihypertensive	10	3.03	
	Blood sugar reducer	7	2.12	
	Thyroid medications	4	1.21	
	Other drugs	32	9.70	
Previous announcement of suicide	I did not announce	89	26.97	0.04
	I announced before taking action	116	35.15	
	I announced after the action	125	37.88	
Time interval from the decision to action	Less than 24 h	192	58.18	<0.0001
	One day to 1 week	34	10.30	
	A week to a month	27	8.18	
	For more than a month	77	23.34	
Child of divorce	No	136	41.21	<0.0001
	Yes	94	28.49	
	Emotional divorce of parents	100	30.30	

Sixty people (18.18%) of those who tried to commit suicide mentioned drug use, of which 34 people (10.30%) had recreational use and 26 people (7.88%) had a drug addiction, and also 151 people (45.76%) consumed alcohol, of which 138 (41.82%) had recreational use and 13 (3.94%) had alcohol addiction (Table 3).

**Table 3:** Frequency of drug and alcohol consumption by gender in suicide attempters referring to hospitals, clinics, and centers affiliated with Jahrom University of Medical Sciences in 2021

Variable	Grouping	Total (%)	Sex		*p* Value^a^
			Male (%)	Female (%)	
		(*n* = 622)	(*n* = 110)	(*n* = 220)	
Alcohol consumption	Recreational consumption	138 (41.82)	79 (71.82)	59 (26.82)	0.013
	Addiction	13 (3.94)	12 (10.91)	1 (0.45)	
Substance use	Recreational consumption	34 (10.3)	28 (25.45)	6 (2.73)	0.089
	Addiction	26 (7.88)	26 (23.64)	0 (0)	

^a^ Fisher exact test, significance level <0.05.

**Figure 2:**
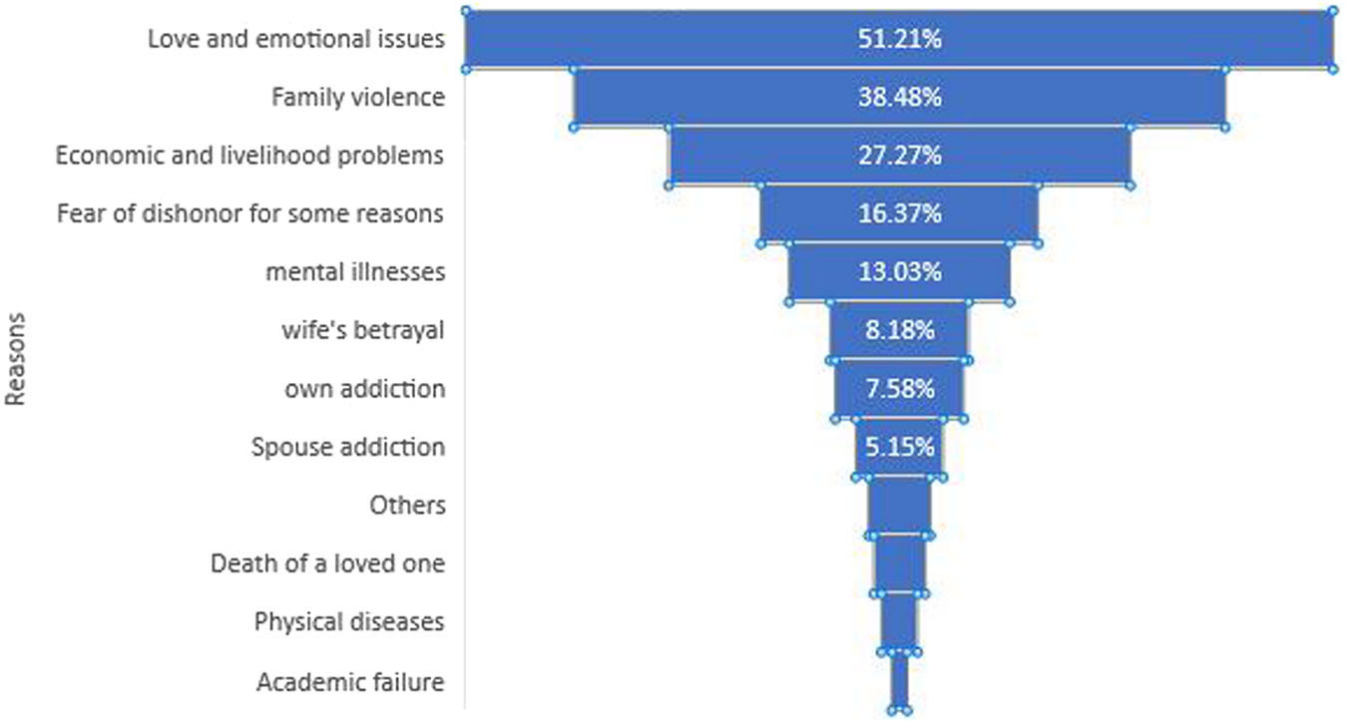
The frequency of causes of suicide attempts among suicide attempters referring to hospitals, clinics, and centers affiliated with Jahrom University of Medical Sciences in 2021

In terms of the cause of suicide attempts, three factors are emotional issues and problems with 169 people (51.21%), family violence—either mental or physical violence—with 127 people (38.48%), and economic issues and problems with 90 people (27.27%). They ranked first to third (Figure 2).

In terms of the method of committing suicide, the highest frequency was drug use by 283 people (85.76%), agricultural poisons (organophosphates) by 16 people (85.4%), and rodenticide death by 11 people (3.3%) (Table 4).

**Table 4:** The distribution of suicide attempt methods among those who refer to hospitals, clinics, and centers affiliated with Jahrom University of Medical Sciences in 2021

Variable	Methods	Frequency	Percentage
Methods of committing suicide	Taking medication	283	85.76
	Use of agricultural pesticides (organophosphates)	16	4.85
	Consumption of rodenticide	11	3.33
	Self‐immolation	1	0.30
	Use of gun	2	0.61
	Taking aluminum phosphide	3	0.91
	Using cutting tools	7	2.12
	Falling from a height	1	0.30
	Hanging	8	2.42
	Others	3	0.91

**Figure 3:**
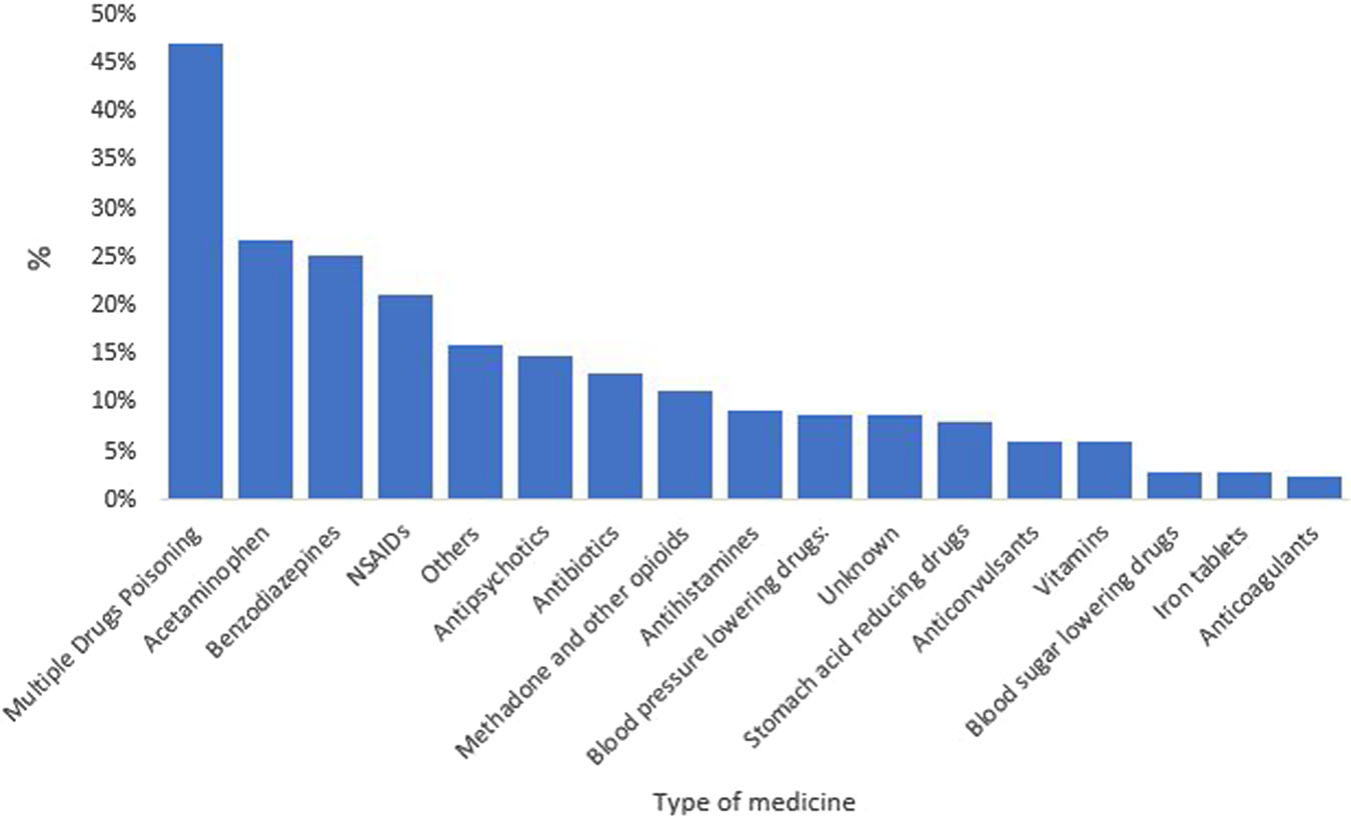
Frequency type of drugs used to attempt suicide in suicide attempters referring to hospitals, clinics, and centers affiliated with Jahrom University of Medical Sciences in 2021

In terms of the type of drug used for suicide, acetaminophen is the most used drug by 76 people (26.86%), followed by benzodiazepines by 71 people (25.09%) and NSAIDs by 60 people (21.20%), respectively. Also, using two or more drugs for suicide is called multiple drugs poisoning; 133 people (47%) of those studied committed suicide with multiple medications (Figure 3).

Finally, 6 cases resulted in death among the people who tried to commit suicide; 4 were men (66.67%), 2 were women (33.33%), 1 was using medicine, and 3 were using aluminum phosphide. Two of them committed suicide by hanging.

## 4 DISCUSSION

This study showed that emotional problems, family violence, and economic problems are among the most important factors of suicide attempts throughout the covid‐19 epidemic. On average, the suicide rate attempts during the study period was calculated as 137 per 100,000 population; however, deaths in completed suicides are small in numbers; it is important to highlight that 50% of deaths are due to highly hazardous pesticides (HHPs) and regulating them will reduce further deaths. Due to the lack of reports in the study area in the years previously the Covid‐19 epidemic, it is possible to compare and examine the impact of this pandemic on its increase or decrease did not have.

Singh,^24^ in a study of suicide cases in some regions of India throughout the lockdown, has shown a twofold rise, and Mishra^3^ reported a fourfold increase in Western Odisha, India, during COVID pandemic. This increase is consistent with hypothesized concepts of stress increased throughout a pandemic because of the fear of contracting COVID‐19, economic problems, and discrimination.^25^, ^26^

Mamun, in a systematic review study in Bangladesh, showed that the prevalence of suicidal ideation during the covid‐19 pandemic period was between 5% and 19.0%, while this rate increased compared to before the start of the pandemic. Important risk factors for suicidal behavior include less knowledge about COVID‐19, lack of preventive COVID‐19 behaviors, higher levels of fear of COVID‐19, living in a highly COVID‐19‐infected region, the infected area had more economic losses due to the pandemic, and experiencing death of relatives or acquaintances by COVID‐19.^15^

Bonsaksen et al.,^16^ in a study of 4527 adults living in Norway during the covid‐19 pandemic, showed that 3.6% reported suicidal thoughts in the past month, compared to 0.2% in the same period before the pandemic. Being in a risk group for complications of COVID‐19 (OR: 2.15, *p* < 0.001) and having economic concerns related to the pandemic (OR: 2.28, *p* < 0.001) were associated with current suicidal thoughts.

Stańdo et al.,^14^ in a study in Poland before and during the COVID‐19 Pandemic, showed an increase in suicide attempts was observed in two age groups of 7−24 and 25−65 years between 2021 and 2019−2020. An increase in suicide was observed among women in all age groups during the study period, while no increase in suicide was observed among men in any age group.

Studies in trends of suicide in high‐income nations showed no increase over previously documented rates. Nevertheless, social, health, political, cultural, and economic scenarios differ from country to country.

According to the reports of the WHO, depression and mental disorders, family violence, rape, sexual abuse, physical diseases, and economic and livelihood problems are among the most important reasons for suicide in different societies.^27^

In a meta‐analysis study in Iran during the years 2001−2014, it was shown that the main reasons for suicide are family and emotional problems, unemployment and poverty, mental illnesses, addiction, and academic failure; the highest frequency was related to family and emotional problems (67.7%).^28^ In another meta‐analysis study on the determination of social factors related to suicide in Iran in 2012, it was shown that the most important social factors affecting are family disputes (30%), marriage problems (26%), economic constraints (12%), and academic failures (5%).^29^ The difference in the causes of suicide in different studies can be due to the culture and climate of the studied people, the time, and the study method. Although family violence has been a critical reason for suicide attempts in the studied subjects, this factor can be considered a strong primary stimulus for suicide attempts, which should be considered for other cultural, economic, and emotional reasons.

Other results showed that drug use had the highest frequency of suicide attempts. Among the drugs used, acetaminophen and benzodiazepines had the highest frequency of use.

In terms of methods of committing suicide, reports in different countries can be various depending on how many people have access to tools and equipment used for suicide.^30^ Lim et al.'s^31^ study in South Korea showed that about 50% of suicide attempters committed suicide using chemical drugs, and the most common method leading to death was using chemical drugs. Bidel et al.,^32^ in a study in the field of the main ways of committing suicide in Iran, it was shown by meta‐analysis in 2012 that using drugs (especially acetaminophen) with a prevalence of 75% is the most important method of committing suicide, which is somewhat close to the results of this study. One of the most important reasons for the high prevalence of suicide attempts by taking drugs is the ease of access to medicines and the lack of strictness of pharmacies in selling drugs without a doctor's prescription, mainly commonly used drugs such as acetaminophen, painkillers, antibiotics, and even benzodiazepines. Furthermore, the people who use these drugs may think they cannot cause serious harm to them. Therefore it is a good option to achieve their goals of suicide—including scaring the people around them and drawing more attention to themselves.

With the continuation of the Covid‐19 pandemic and its rapid spread worldwide, one of the strategies to control it was to set a time as quarantine to isolate the sick person until the time of transmission to others.^33^ Some studies conducted in this field also considered quarantining patients as one of the appropriate solutions to deal with this pandemic.^34^, ^35^ On the other hand, quarantine can cause relative isolation of the sick person from the community and the surrounding environment. It can increase psychological, physical, and interpersonal problems in a range of anxiety, despair, depression, interpersonal conflicts, and fear of disease and death in patients.^36^, ^37^ To acknowledge the link between depression related to pandemic factors, including quarantine and other containment measures, and weight gain, the term “depreobesity” was created.^38^, ^39^

Other results showed that depression is the most common disease among people attempting suicide; on the other hand, among 164 patients with depression, only 9.7% of these people were treated with antidepressants.

Studies conducted in countries such as America,^40^ Australia,^41^ England,^42^ France,^43^ and China^44^ have reported that depression is the most critical risk factor for suicide attempts. A meta‐analysis study by May et al.^45^ reported that most suicidal ideators do not commit suicide. Therefore, it is helpful to understand what differentiates suicide attempters from suicidal thinkers.

The results showed that alcohol and drug use, depression, anxiety, posttraumatic stress disorder, and history of sexual abuse have increased in people taking action compared to ideators.^45^ Another study has described that about 15% of individuals with depression eventually commit suicide.^46^

Many studies have been conducted on the effect of antidepressants in treating depression and preventing suicide, and completely different results have been extracted. Some studies have reported a positive effect,^47^, ^48^, ^49^ and others have said an adverse effect or lack of effectiveness of these drugs.^50^, ^51^, ^52^

One of the reasons for the non‐use of antidepressants by the participants may be the belief that these drugs cause dependence and addiction and should be taken for a lifetime. Moreover, the expectation of the rapid effect of these drugs on the treatment of depression in the view of the patients had caused them to stop taking their medications after a short time due to not receiving the expected therapeutic response. Therefore, it is recommended that therapists, in the case of prescribing antidepressants, provide more detailed training and information for their patients regarding the above issues.

Other findings showed that the frequency of alcohol consumption among the participants was 45.76%. Several studies have shown alcohol consumption as an important risk factor in increasing the probability of committing suicide in people prone to suicide.^53^, ^54^, ^55^, ^56^

In a meta‐analysis study in Iran in 2014, Darvishi et al. showed that alcohol use disorder meaningfully raises the risk of suicidal thoughts, attempted suicide, and complete suicide can be a significant prophesier of suicide.^57^

In their study, Kendall^58^ showed that some suicide attempters consumed alcohol shortly before suicide to boost their courage. They reported that alcohol and its dependence often lead to decline, social isolation, self‐esteem loss, and thus depression. These psychological changes are among the predisposing factors for suicide attempts.

One of the reasons for people's tendency to drink alcohol, especially in young age groups, may be that drinking alcohol in friendly groups gives them a false sense of self‐confidence and growth, and they consider it wrong to use alcohol, cigarettes, or sometimes use drugs to be different from their peers. Therefore, since alcohol consumption is one of the risk factors in people prone to depression and suicide, therapists must provide more information about the harms of its consumption to the general public, especially to nervous and mental patients and suicide attempters.

Unlike a study in the central province that showed a higher proportion of suicides at the diploma and higher education level,^59^ in this study, most of the suicide attempters had a secondary and high‐school education. Similar findings have been reported in other studies conducted in different regions of Iran.^60^, ^61^, ^62^

Although this conclusion is based on the observed frequency, it seems that higher education can improve a person's use of coping methods and help him in daily crises.

Other findings of the present study showed that the frequency of suicide attempts is higher in urban residents, which is consistent with the results of other studies.^63^, ^64^ The higher rate of suicide in urban communities can be due to different lifestyles, more elevated amount and intensity of stress, and less social dependence.

In this study, nearly 35% of people had a history of suicide attempts. According to the WHO and some studies report, the most important risk factor for suicide is having a previous history of suicide attempts.^4^, ^15^, ^16^ Therefore, it is necessary for people who commit suicide not to be left to their own devices after being discharged from the hospital and to be under the supervision of mental health support institutions to take the necessary intervention measures to prevent the next suicide attempt.

The present study showed that 14.85% of suicide attempters had borderline personality disorder. Several studies have reported the relationship between suicide attempts and personality disorders.^65^, ^66^, ^67^

In a meta‐analysis study, Pompili et al.^68^ showed that suicide was the leading cause of death among patients with borderline personality disorder. From 1980 to 2005, on average, 100,000 suicides leading to death worldwide were caused by people with borderline personality disorder.

People with borderline personality disorder experience rapid changes in mood, and their behavior is very unpredictable. These are dependent people, but they may lose their friends frequently due to frequent anger and lack of anger control. These people can't stand being alone, so they frantically search for a new relationship, and despite their strong expression of emotional states, they often feel depressed. For this reason, the chance of suicide in them is higher than in general society.^65^, ^68^

Other findings of this study showed that 28.49% of respondents were children of divorce, and 30.30% reported emotional divorce among their parents. The results of a survey in Isfahan in 2020 on teenagers and young people from families on the verge of separation indicated that the tendency of teenagers and young people from families on the verge of divorce to commit suicide was significantly higher than ordinary people.^69^ In their study in Germany, Hardt et al.^70^ showed that parental divorce raises the relative risk of suicide by 1.6 in children of divorce.

There were some limitations in our study. First, this study was conducted cross‐sectionally and in 1 year; therefore, it was impossible to evaluate the change process and investigate the causal relationships between the influencing factors. Second, this study is not considering all suicide attempters in the study place because of the reluctance of some of them to participate in the study because of fear of revealing their secrets despite being assured by the researcher, lack of access to some attempters due to discharge with the consent of the patients in the early hours of referring the hospital emergency room and the lack of cooperation of some relatives of the deceased patients due to grief or lack of sufficient knowledge of the cause of the deceased person's suicide attempt, which can affect the results.

## 5 CONCLUSION

The most critical suicide factors throughout the covid‐19 epidemic were emotional, marital incompatibility, and economic issues. Drug use was the most important method of committing suicide. In times of crisis, a multisectoral public health approach is needed to prevent increased anxiety, stress, and subsequent suicide attempts. Since, in the case of complete suicide, 50% of deaths are due to HHPs, their attention and regulation will reduce even more deaths.

A risk communication strategy is recommended in communities to rapidly disseminate accurate and preventive information to reduce public concern about outbreaks and teach how to change behavior to mitigate anxiety and stress.

For future studies, it is suggested to use more accurate statistical methods, such as interrupted time‐series analysis, to investigate the suicide trend previously and after the covid‐19 epidemic and to investigate its effect on the increase or decrease of suicide in more detail to be placed.

## AUTHOR CONTRIBUTIONS

**Mina Sefid Fard Jahromi**: conceptualization; data curation; investigation; project administration; supervision; validation; visualization; writing – original draft. **Mohammad Hadi Eghbal**: data curation; formal analysis; investigation; resources; software; visualization; writing – original draft. **Vahid Rahmanian**: conceptualization; data curation; formal analysis; methodology; project administration; validation; writing – original draft; writing – review & editing.

## CONFLICT OF INTEREST

The authors declare no conflict of interest.

### ETHICS STATEMENT

The ethical considerations, such as explaining the study's objectives, voluntary participation, the confidentiality of the collected information, and obtaining informed consent, were fully observed in this study. The Research Ethics Committee of Jahrom University of Medical Sciences approved the study protocol (ID IR.JUMS.REC.1399.146.)

## TRANSPARENCY STATEMENT

The lead author Vahid Rahmanian affirms that this manuscript is an honest, accurate, and transparent account of the study being reported; that no important aspects of the study have been omitted; and that any discrepancies from the study as planned (and, if relevant, registered) have been explained.

## Supporting information

Supporting information.

## Data Availability

The authors acknowledge that data supporting the findings of this study are available in the article [and/or] its supplementary material.
