# Supplementary material for: Epidemiology of suicide and suicide attempts in Jahrom district, Southern Iran in light of COVID pandemic: A prospective observational study
Source: Health Sci Rep. 2022 Nov 14;5(6):e933. doi: 10.1002/hsr2.933 (PMC9662693; doi:10.1002/hsr2.933)
Supplement: Supplementary file 1 — Supporting information. [file HSR2-5-e933-s001.docx]

Supplementary table1. Crude and age-standardized suicide rates for all ages(per 100 000) in males and females in WHO Eastern Mediterranean Region in 2019. (Source: WHO)^4^

| Country | Sex | Number of  suicides, all  ages, 2019 | Crude suicide  rates, all ages  (per 100 000),  2019 | Age-standardized  suicide rates,  all ages  (per 100 000),  2019 |
| --- | --- | --- | --- | --- |
| Afghanistan | both sexes | 1573 | 4.1 | 6.0 |
|  | females | 668 | 3.6 | 5.7 |
|  | males | 905 | 4.6 | 6.2 |
| Bahrain | both sexes | 145 | 8.9 | 7.2 |
|  | females | 14 | 2.4 | 2.3 |
|  | males | 132 | 12.5 | 9.9 |
| Djibouti | both sexes | 94 | 9.6 | 11.9 |
|  | females | 29 | 6.3 | 7.6 |
|  | males | 65 | 12.6 | 16.3 |
| Egypt | both sexes | 3022 | 3.0 | 3.4 |
|  | females | 995 | 2.0 | 2.2 |
|  | males | 2027 | 4.0 | 4.6 |
| Iran (Islamic Republic  of) | both sexes | 4334 | 5.2 | 5.1 |
|  | females | 1128 | 2.7 | 2.7 |
|  | males | 3206 | 7.7 | 7.5 |
| Iraq | both sexes | 1418 | 3.6 | 4.7 |
|  | females | 378 | 1.9 | 2.4 |
|  | males | 1040 | 5.2 | 7.3 |
| Jordan | both sexes | 165 | 1.6 | 2.0 |
|  | females | 37 | 0.7 | 0.9 |
|  | males | 128 | 2.5 | 3.0 |
| Kuwait | both sexes | 122 | 2.9 | 2.7 |
|  | females | 12 | 0.7 | 0.7 |
|  | males | 110 | 4.3 | 3.8 |
| Lebanon | both sexes | 190 | 2.8 | 2.8 |
|  | females | 59 | 1.7 | 1.7 |
|  | males | 131 | 3.8 | 3.9 |
| Libya | both sexes | 304 | 4.5 | 4.5 |
|  | females | 98 | 2.9 | 2.9 |
|  | males | 206 | 6.0 | 6.1 |
| Morocco | both sexes | 2617 | 7.2 | 7.3 |
|  | females | 865 | 4.7 | 4.7 |
|  | males | 1752 | 9.7 | 10.1 |
| Oman | both sexes | 241 | 4.9 | 4.5 |
|  | females | 17 | 1.0 | 1.1 |
|  | males | 224 | 6.8 | 6.4 |
| Pakistan | both sexes | 19331 | 8.9 | 9.8 |
|  | females | 4560 | 4.3 | 4.7 |
|  | males | 14771 | 13.3 | 14.6 |
| Qatar | both sexes | 165 | 5.8 | 4.7 |
|  | females | 12 | 1.7 | 1.7 |
|  | males | 153 | 7.2 | 5.7 |
| Saudi Arabia | both sexes | 2046 | 6.0 | 5.4 |
|  | females | 282 | 1.9 | 1.9 |
|  | males | 1764 | 8.9 | 7.8 |
| Somalia | both sexes | 1219 | 7.9 | 14.7 |
|  | females | 293 | 3.8 | 7.1 |
|  | males | 926 | 12.0 | 22.8 |
| Sudan | both sexes | 1644 | 3.8 | 4.8 |
|  | females | 590 | 2.8 | 3.3 |
|  | males | 1054 | 4.9 | 6.3 |
| Syrian Arab Republic | both sexes | 333 | 1.9 | 2.1 |
|  | females | 59 | 0.7 | 0.8 |
|  | males | 273 | 3.2 | 3.5 |
| Tunisia | both sexes | 383 | 3.3 | 3.2 |
|  | females | 113 | 1.9 | 1.8 |
|  | males | 270 | 4.7 | 4.6 |
| United Arab Emirates | both sexes | 628 | 6.4 | 5.2 |
|  | females | 89 | 3.0 | 2.6 |
|  | males | 539 | 8.0 | 6.3 |
| Yemen | both sexes | 1699 | 5.8 | 7.1 |
|  | females | 672 | 4.6 | 5.3 |
|  | males | 1026 | 7.0 | 9.0 |
